# Total Intramuscular Fat Fraction of Thigh Muscles as a Predictor of Nusinersen Efficacy in Pediatric SMA Type II and III

**DOI:** 10.3390/diagnostics15060753

**Published:** 2025-03-17

**Authors:** Kiiko Iketani, Hiroyuki Awano, Hiromi Hashimura, Shoko Sonehara, Hiroaki Hanafusa, Yoshinori Nambu, Hisahide Nishio, Kandai Nozu, Ryosuke Bo

**Affiliations:** 1 Department of Pediatrics, Kobe University Graduate School of Medicine, 7-5-1, Kusunoki-cho, Chuo-ku, Kobe 650-0017, Japan; kiiko@med.kobe-u.ac.jp (K.I.); sonesho@med.kobe-u.ac.jp (S.S.); hhiroaki@med.kobe-u.ac.jp (H.H.); ynambu@med.kobe-u.ac.jp (Y.N.); nozu@med.kobe-u.ac.jp (K.N.); ryobo@med.kobe-u.ac.jp (R.B.); 2 Research Initiative Center, Organization for Research Initiative and Promotion, Tottori University, 86 Nishi-cho, Yonago 683-8503, Japan; 3 Department of Radiology, Kobe University Graduate School of Medicine, 7-5-1, Kusunoki-cho, Chuo-ku, Kobe 650-0017, Japan; h.hashimura@hotmail.co.jp; 4 Faculty of Rehabilitation, Kobe Gakuin University, 518 Arise, Ikawadani-cho, Nishi-ku, Kobe 651-2180, Japan; nishio@reha.kobegakuin.ac.jp

**Keywords:** spinal muscular atrophy, muscle magnetic resonance imaging, nusinersen, lipid metabolism, biomarker

## Abstract

**Background/Objectives**: Nusinersen is a disease-modifying drug for spinal muscular atrophy (SMA) that improves motor function. However, its effects on the skeletal muscles remain unclear. This study aimed to assess the intramuscular fat fraction in patients with SMA types II and III using muscle magnetic resonance imaging (MRI) and to explore the relationship between muscle tissue, lipid metabolism, and motor function during nusinersen treatment. **Methods**: This study included seven pediatric patients with SMA types II and III who received nusinersen treatment. Muscle MRIs were performed at three time points. Images of the central thigh were used to measure the cross-sectional area (CSA) and muscle fat area, and the intramuscular fat fraction (IMFF) was calculated. The thigh muscles were categorized into three groups: quadriceps, adductor, and hamstrings. **Results**: The median (range) of total IMFF for SMA type II and III at T-0, T-2, and T-4 were 18.5 (12.6–48.4), 24.4 (10.1–61.4), and 39.0 (30.0–68.6) % and increased over time. In five patients whose motor function was evaluated, a moderate negative correlation was observed between the changes in the Hammersmith Functional Motor Score Expanded (HSFME) and IMFF (r = −0.51). No significant changes in serum triglyceride or total cholesterol levels were observed during treatment. **Conclusions**: An increase in IMFF was associated with a decline in motor function. The baseline IMFF score was related to improvements in motor function scores, suggesting that the IMFF of the thigh muscle may serve as a novel, objective, and quantitative skeletal muscle-related biomarker for predicting the effects of nusinersen on muscle tissue.

## 1. Introduction

Spinal muscular atrophy (SMA) is an inherited lower motor neuron disease caused by abnormalities in *SMN1*, with progressive muscle atrophy as its main clinical feature [1]. SMA is classified into five subtypes based on phenotype [2]. SMA type 0 is the most severe form, with onset in utero and severe respiratory distress after birth. Type I (Werdnig-Hoffmann disease) is a severe form in which the patient does not achieve the ability to sit independently, with onset within 6 months of birth. Type II (Dubowitz disease) represents an intermediate form in which patients can sit without support but do not achieve the ability to stand or walk, with an onset within 18 months of age. Type III is a milder form, wherein patients are able to stand and walk and develops after 18 months of age. Type IV is the mildest form, with onset occurring after 30 years of age.

*SMN2* is a gene homologous to *SMN1*, located on the centromeric side of *SMN1* on the long arm of chromosome 5. Although *SMN2* also produces full-length SMN proteins, minor differences in the nucleotide sequence alter splicing [3,4], resulting in a lower capacity for SMN protein production [5]. Although *SMN2* cannot fully compensate for SMN protein production from *SMN1*, the copy number of *SMN2* is associated with disease severity in patients with SMA [2]. The copy number of *SMN2* is inversely proportional to clinical severity.

Nusinersen is an antisense oligonucleotide drug that modifies the splicing of *SMN2* exon 7, thereby increasing the production of full-length SMN proteins. Clinical trials in infants and children with SMA types I–III have shown improvements in motor function scores, including the Hammersmith Infant Neurological Examination Section 2 (HINE2) and Hammersmith Expanded Functional Motor Scale (HSFME) [6,7]. While these improvements are likely due to the beneficial effects on skeletal muscles, the direct impact of nusinersen on SMA skeletal muscles remains unknown.

Magnetic resonance imaging (MRI) is a non-invasive imaging modality that provides objective assessments and is the gold standard for evaluating muscle mass and quality [8]. Muscle MRI has been reported as a predictive or efficacy marker for hereditary muscular disorders, such as Duchenne muscular dystrophy and limb-girdle muscular dystrophy [9].

This study aimed to evaluate intramuscular fat in seven patients with SMA types II and III undergoing nusinersen treatment over four years using muscle MRI and to investigate the relationship between muscle tissue, fat metabolism, and motor function. We also examined whether muscle MRI findings could be used as predictive or monitoring biomarkers of nusinersen treatment.

## 2. Materials and Methods

### 2.1. Cases

The study included seven patients with SMA types II and III who visited Kobe University Hospital and began nusinersen treatment between January 2018 and January 2019. The diagnosis of SMA was confirmed by identifying *SMN1* gene variants. Nusinersen was administered according to the Japanese package insert (https://pins.japic.or.jp/pdf/newPINS/00066991.pdf) (accessed on 9 December 2024). Specifically, Nusinersen was administered intrathecally three times during the initial loading period of three months and then every six months during the maintenance period.

### 2.2. Muscle MRI

MRI examinations of the entire femur were conducted at baseline (T-0), two years (T-2), and four years (T-4) after the initiation of treatment using a 1.5 Tesla MR system (SIGNA™ Voyager, GE HealthCare, Chicago, IL, USA). The imaging protocol included STIR and T1-weighted sequences in both the coronal and axial planes, as well as T2-weighted images in the transverse plane. No contrast agents were administered. The typical imaging parameters of T1 weighted axial images used for analysis were as follows: field of view, 360 × 360 mm; matrix, 400 × 320; spatial resolution, 0.9 × 1.1 mm; repetition time/echo time, 650/8.3 msec; slice thickness, 5 mm; slice gap 2 mm; slices 38; parallel imaging/factor, Generalized Auto calibrating Partially Parallel Acquisition (GRAPPA)/1.5; echo train length, 3; number of excitation, 1; scan time, 1 min 20 s.

Midfemoral slices on T1-weighted axial images were used for the analysis. Using analysis software (Ziostation2, Ziosoft Inc., Tokyo, Japan), the boundaries of the 12 muscles were manually traced: the lateral vastus, medial vastus, medial rectus femoris, rectus femoris, gracilis, sartorius, adductor longus, adductor magnus, semimembranosus, semitendinosus, long head of the biceps femoris, and short head of the biceps femoris. These regions of interest (ROI) were determined by agreement between an experienced pediatrician (KI with 6 years of experience) and a radiologist (HH with 21 years of experience) (Supplementary Figure S1A).

A signal intensity threshold was set for each case to determine the areas of the muscle and adipose tissue in the ROIs. Those with values above the threshold were classified as adipose tissue, while those with values below the threshold were classified as muscle (Supplementary Figure S1B).

The cross-sectional area (CSA) and percentage of intramuscular fat fraction (IMFF) were determined for each thigh muscle. IMFF was calculated by dividing the fat area within the ROI by the CSA. The total CSA and IMFF were the sum of the values of each muscle. The muscle groups were classified as follows: (1) quadriceps group: lateral vastus, medial vastus, medial rectus femoris, and rectus femoris; (2) adductor group: gracilis, sartorius, adductor longus, and adductor magnus; and (3) hamstring group: semimembranosus, semitendinosus, long head of the biceps femoris, and short head of the biceps femoris.

### 2.3. Clinical Data

The background characteristics, blood test results, and motor function scores of the patients were obtained from their medical records. Motor function was assessed using the Hammersmith Functional Motor Scale Expanded (HFMSE). For two cases with HFMSE scores of 2 points or less at T-0, the Children’s Hospital of Philadelphia Infant Test of Neuromuscular Disorders (CHOP-INTEND) was used to evaluate the motor function.

### 2.4. Statistics

The Shapiro–Wilk test was used to determine whether the dataset followed a normal distribution. If the *p*-value was less than 0.05, the dataset was considered non-normally distributed. Student’s *t*-test was used for comparisons between the two groups if the data followed a normal distribution; otherwise, the Mann–Whitney U test was used. For comparisons among three or more groups, Tukey’s or Dunn’s multiple comparison tests were used.

Correlations were analyzed using Pearson’s correlation coefficient or Spearman’s rank correlation coefficient. Correlation strength was categorized as follows: |r| < 0.4 (weak), 0.4–0.7 (moderate), and >0.7 (strong). Statistical significance was set at *p* < 0.05. All statistical analyses, except for effect size, were performed using GraphPad Prism ver. 10.4.0 (GraphPad Software, Boston, MA, USA). Effect size (d value) was calculated using Excel ver. 2502 (Microsoft, Redmond, WA, USA). d = 0.2, 0.5, 0.8, and 1.2 were classified as small, medium, large, and very large, respectively [10].

### 2.5. Ethics

Written informed consent for MRI was obtained from all patients and their parents. The study was approved by the Ethics Committee of Kobe University Graduate School of Medicine (No. B200086; approved on 24 June 2020).

## 3. Results

### 3.1. Patients

The background characteristics of the patients are summarized in Table 1. The study included five patients with SMA type II (four boys and one girl) and two patients with SMA type III (two girls). All patients had three copies of *SMN2*. One patient with SMA type II underwent gastrostomy and used non-invasive positive pressure ventilation (NPPV) only at night. At T-0, the median (minimum–maximum) ages for SMA type II and type III patients were 6 years and 2 months (3 years 1 month–9 years 8 months) and 4 years and 2 months (3 years 4 months–4 years 11 months), respectively. The median number of nusinersen treatments by T-4 was 12 for SMA type II patients and 10.5 times for SMA type III patients. The BMI of all patients was <18.5 kg/m^2^ at T-0, indicating an underweight status, which persisted in six patients (86%) at T-4.

**Figure 1:**
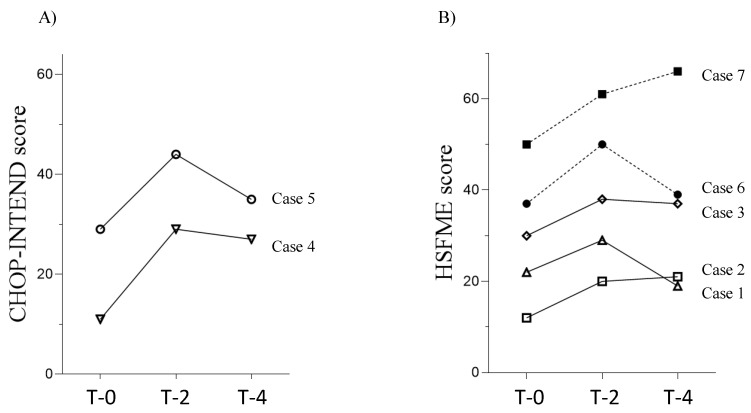
Changes in motor function scores in patients with SMA types II and III. (**A**) Changes in the CHOP-INTEND scores of two patients with SMA type II. (**B**) Changes in HSFME scores for three patients with SMA type II and two patients with SMA type III. Open symbols represent patients with SMA type II, and filled symbols represent patients with SMA type III.

Figure 1A,B shows the changes in motor function scores. Motor function was assessed using the CHOP-INTEND in two patients and the HFMSE in five patients. Four of the five patients with SMA type II showed an improvement in CHOP-INTEND or HFMSE scores at T-2 and maintained these scores until T-4. In Case 1, with SMA type II, the HFMSE score at T-4 was below the baseline. Among patients with SMA type III, the HFMSE score of Case 7 showed a steady increase, whereas the score initially improved at T-2 but declined at T-4 in Case 6.

### 3.2. Changes in CSA of the Thigh Muscles

**Figure 2:**
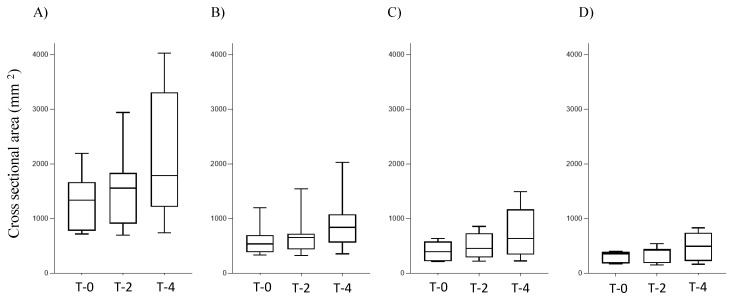
Changes in CSA during nusinersen treatment. (**A**) total CSA, (**B**) quadriceps group, (**C**) adductor group, and (**D**) hamstring group.

The median (minimum-max) total CSA for SMA type II and III patients at T-0, T-2, and T-4 were 1338 (717–2193), 1556 (695–2940), and 1787 (741–4029) mm^2^, respectively (Figure 2A). There was a large magnitude of difference in the median total CSA between T-0 and T-4 (d = 1.04); however, no significant differences were observed between the time points (*p* = 0.18). Time changes in the CSA of the quadriceps, adductors, and hamstrings are shown in Figure 2B–D. Although the median CSA increased in all muscle groups during treatment (d = 0.78, 1.17, and 0.97 for the quadriceps, adductor, and hamstring groups, respectively, between T-0 and T-4), the changes were not statistically significant.

### 3.3. IMFF in Type II and III Patients

**Figure 3:**
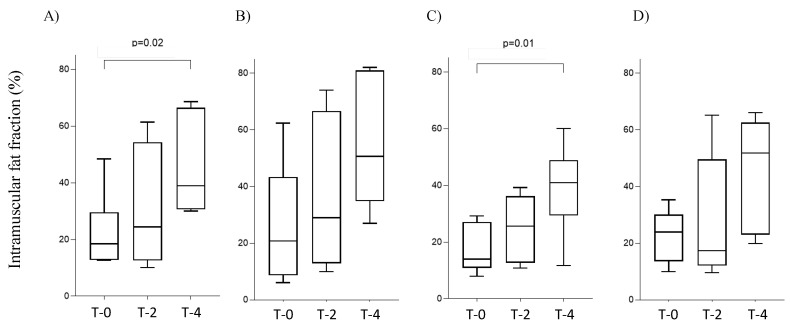
Changes in IMFF during nusinersen treatment. (**A**) total IMFF, (**B**) quadriceps group, (**C**) adductor group, (**D**) hamstring group.

The median (range) of total IMFF at T-0, T-2, and T-4 were 18.5 (12.6–48.4), 24.4 (10.1–61.4), and 39.0 (30.0–68.6) % (Figure 3A). Compared to T-0, the IMFF at T-4 showed a significant increase (*p* = 0.02, d = 1.82). The IMFF values of the quadriceps and adductors increased over time (d = 1.56, 1.52, respectively, between T-0 and T-4) (Figure 3B,C). The IMFF of the hamstrings decreased at T-2 but increased at T-4 (Figure 3D). The IMFF of the adductors showed a significant increase at T-4 compared to that at T-0 (*p* = 0.01, d = 1.81).

### 3.4. Motor Function Scores and IMFF

**Figure 4:**
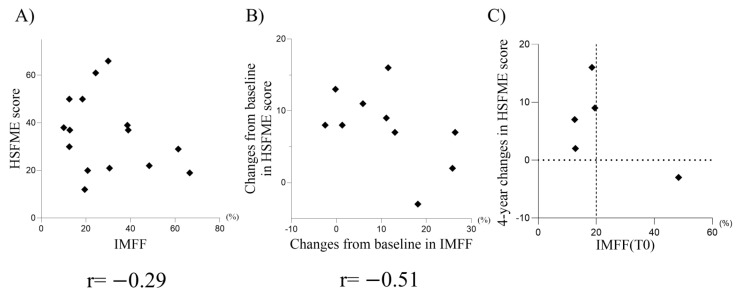
Relationship between HSFME scores and IMFF. (**A**) Correlation between HSFME scores and IMFF. (**B**) Correlation between changes in HSFME scores and changes in IMFF from T-0. (**C**) Correlation between IMFF and changes in HSFME scores over four years. The vertical dotted line indicates an IMFF of 20%, and the horizontal dotted line indicates no change (0). r: correlation coefficient.

For the two patients with SMA type II evaluated using CHOP-INTEND, no strong correlation was observed between the motor function scores and IMFF (r = 0.03). In five patients (three with type II and two with type III) assessed using the HFMSE, a weak negative correlation was found between HFMSE scores and IMFF at T-0, T-2, and T-4 (r = −0.29; Figure 4A).

Changes in the HFMSE scores and IMFF from T-0 to T-2 and T-4 were calculated and analyzed. A moderate negative correlation was observed between changes in HFMSE scores and IMFF (r = −0.51, Figure 4B). Among the four patients whose HFMSE scores increased from T-0 to T-4 (Cases 1, 3, 6, and 7), the total IMFF at T-0 was below 20%, whereas the single patient (Case 2) whose HFMSE scores decreased had a high ratio of total IMFF of 48.4% at T-0 (Figure 4C).

### 3.5. Lipid Metabolism

**Figure 5:**
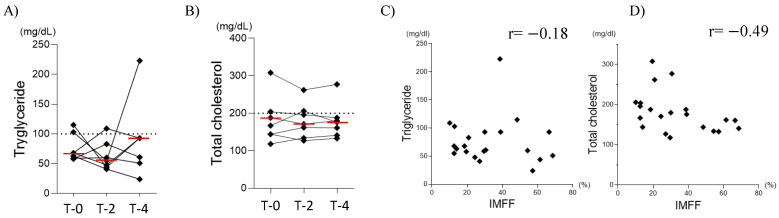
Lipid metabolism during nusinersen treatment. (**A**) Changes in serum triglyceride levels, (**B**) Changes in serum total cholesterol levels, (**C**) Correlation between serum triglycerides and IMFF, and (**D**) Correlation between serum total cholesterol and IMFF. The red horizontal lines represent median values.

Three patients with SMA showed high triglyceride (TG) levels (≥100 mg/dL) [11] during the four years (Figure 5A), but no patient had persistently elevated TG levels. Three patients exhibited high total cholesterol (TC) levels (≥200 mg/dL) [11] during the four years (Figure 5B). One patient with SMA type II (Case 2) had persistently elevated TC levels.

No significant changes or large differences were observed in TG or TC levels after nusinersen treatment (d = 0.33 and 0.05 for TG and TC, respectively, between T-0 and T-4) (Figure 5A,B). A weak negative correlation was observed between IMFF and serum triglyceride levels (r = −0.18; Figure 5C). A moderate negative correlation was observed between IMFF and total cholesterol levels (r = −0.49, Figure 5D). When examining the correlations between the IMFF in different muscle groups and body composition or lipid metabolism, moderate negative correlations were noted between the serum total cholesterol levels and the quadriceps and hamstrings (r = −0.48 and r = −0.48, respectively) (see Supplementary Table S1).

## 4. Discussion

### 4.1. Total IMFF of the Thigh as a Marker of Disease Progression

We report four-year changes in the IMFF of the thigh muscles in seven pediatric patients with SMA types II and III who underwent nusinersen treatment. The IMFF increased in all muscle groups over the four years. In five patients evaluated using the HFMSE, a moderate correlation was observed between changes in the IMFF and HFMSE scores, suggesting that IMFF changes are associated with disease progression.

Since the 1990s, muscle MRI studies of SMA have shown muscle atrophy and increased intramuscular fat [12,13,14]. Qualitative muscle MRI findings have garnered attention as potential markers for diagnosing and monitoring SMA. Subsequent studies have examined the relationship between quantitative assessments of muscle volume, fat fraction, and clinical data, revealing that a decrease in muscle mass and an increase in intramuscular fat negatively impact motor function [15,16]. Intramuscular fat accumulation progresses with age and disease progression [13,17]. In the future, it would be desirable to compare these data on natural history control with those for the nusinersen treatment group to evaluate its potential usefulness as a treatment-monitoring marker.

Additionally, the IMFF should be evaluated as a monitoring marker for therapies targeting the skeletal muscles of SMA, including myostatin inhibitors [18].

### 4.2. Baseline Total IMFF of the Thigh as a Predictor of Treatment Response

All four patients whose HFMSE scores improved at T-4 had a total IMFF < 20% at T-0. In contrast, the total IMFF of the patient whose HFMSE score declined was above 20%, suggesting that the total IMFF could serve as a predictor of nusinersen treatment efficacy. Otto et al. also reported that the average pretreatment IMFF was lower in patients whose HFMSE scores improved [19]. Shimizu-Motohashi et al. qualitatively assessed pretreatment muscle MRI using Mercuri and Atrophy grades in patients with SMA types II and III and found a negative correlation between pretreatment fat replacement/muscle atrophy and HFMSE scores 15 months after treatment [20]. These findings suggest that pretreatment IMFF may predict motor function outcomes after nusinersen therapy.

Moreover, it was reported that longer disease duration before treatment correlates with less improvement in motor function scores, and older age at treatment affects nusinersen efficacy [6,7,21]. As intramuscular fat increases with age [19], these data may reflect a relationship between fat replacement and motor function scores.

Recently, neonatal mass screening for SMA has been implemented in several countries to identify newborns or infants with very short disease durations before treatment initiation [22,23,24]. Patients who received treatment before symptom onset generally had better motor outcomes than those who received treatment after the symptom onset. However, there are individual differences in the changes in HFMSE scores and achievement of motor milestones [25]. Investigating the association between IMFF and motor function in these patients is essential for further exploring the utility of IMFF as a predictive marker of treatment response.

### 4.3. Does Metabolic Abnormality in SMA Relate to IMFF?

SMA is associated with altered lipid metabolism [26,27]. Adult patients with untreated SMA type III treated with disease-modifying drugs show a higher prevalence of dyslipidemia, including hyper-LDL cholesterolemia, hypo-HDL cholesterolemia, and hyper-TC, than the general population. In our pediatric cohort, three patients showed elevated TG and TC levels during the study period. Notably, one patient (case 2) displayed persistently high TC levels. These findings suggest that SMN protein deficiency may affect lipid metabolism not only in peripheral nerves but also in the liver and muscles. Supporting this, SMA model mice exhibit non-alcoholic fatty liver disease (NAFLD) phenotypes [28], and pathological samples from patients with SMA confirm hepatic steatosis [26].

Although the effects of nusinersen treatment were initially expected to be confined to the nervous system, we investigated its potential influence on systemic lipid metabolism. In our study, nusinersen treatment had no significant effect on serum TC or TG levels. Furthermore, a negative correlation was observed between serum TC and IMFF levels, suggesting that there is no direct link between systemic lipid metabolism and IMFF-related fat metabolism.

### 4.4. Mechanisms Behind IMFF in SMA

The mechanisms underlying increased IMFF in the SMA remain unclear. In healthy individuals, intramuscular fat increases with disuse and age [29,30]. Patients with deteriorating SMA experience an increase in IMFF due to aging and disuse [13,17], similar to healthy individuals. However, it is difficult to determine whether intramuscular fat is due to aging or a lack of exercise. This is because it is clearly linked to inactivity in both healthy young individuals and those with muscle disorders, with accumulation occurring after just four weeks of immobilization [31]. Moreover, it is unclear whether SMN protein deficiency or nusinersen treatment itself causes or accelerates the increase in intramuscular fat.

Wang et al. recently reported the involvement of various signaling systems in IMFF formation [32]. Furthermore, SMN has been implicated in processes such as myotube formation and calcium signaling [33,34]. Therefore, reduced SMN protein levels in skeletal muscles may activate the signaling pathways related to IMFF formation. The effect of nusinersen treatment on intramuscular fat cannot be evaluated due to the lack of a study that directly compares the treatment group with the non-treatment group.

Systemic therapies, including risdiplam and onasemnogene abeparvovec, may alter intramuscular lipid metabolism. Further studies are necessary to explore their relationship with IMFF.

### 4.5. Study Limitations

This study had limitations. First, the research involved a small cohort of seven participants, and due to the limited sample size, careful consideration is required when applying the results to a broader population. Future studies, such as multi-center collaborative research, are needed to confirm the validity of the results using larger sample sizes. Second, dietary habits were not considered, and factors other than nusinersen may have influenced IMFF and lipid metabolism. Additionally, while they received medical management at the same facility, differences in the age of initiation of treatment and differences in rehabilitation frequency and intensity may have influenced our results. Third, while a consensus-based approach to ROI delineation was employed to minimize subjective bias and enhance data consistency, the potential for inter-rater variability in tracing results, even after consensus, cannot be eliminated. Finally, the study did not compare the results with electrophysiological data, such as motor unit number estimation or compound muscle action potential. The relationship between electrophysiological changes and IMFF remains unclear.

## 5. Conclusions

The total IMFF of the thigh muscles before the initiation of nusinersen treatment has been shown to be a potential predictive marker of treatment efficacy. Moreover, the negative correlation between the changes in the total IMFF and HFMSE scores revealed that an increase in the IMFF was associated with a decline in motor function, suggesting that IMFF of the thigh muscles may serve as a novel, objective, and quantitative skeletal muscle-related biomarker reflecting the pathology of SMA.

## Figures and Tables

**Table 1:** Characteristics of patients with spinal muscular atrophy.

Case	1	2	3	4	5	6	7
Type	II	II	II	II	II	III	III
Sex	Female	Male	Male	Male	Male	Female	Female
*SMN2* copy number	3	3	3	3	3	3	3
Age at onset	2 y 0 m	9 m	1 y 8 m	9 m	10 m	1 y 10 m	1 y 3 m
Nutritional support	None	None	None	Gastrostomy tube	None	None	None
NPPV	None	None	None	Nighttime only	None	None	None
Age at initiation of nusinersen	9y8m	6y3m	3y1m	6y2m	4y6m	3y4m	4y11m
Number of nusinersen treatment	12	12	12	12	11	10	11
BMI (SDS)							
T0	12.8 (−2.5)	12.4 (−3.0)	15.8 (0.3)	9.0 (−6.6)	11.3 (−3.5)	14.4 (−1.0)	15.5 (−1.7)
T4	17.1 (−1.1)	13.3 (−2.4)	17.7 (0.4)	8.3 (−9.6)	13.1 (−2.3)	21.4 (1.7)	15.8 (−1.5)

## Data Availability

The raw data supporting the conclusions of this article will be made available by the authors upon reasonable request.

## Supplementary Materials

The following supporting information can be downloaded at: https://www.mdpi.com/article/10.3390/diagnostics15060753/s1, Figure S1: Cross-sectional images of a thigh MRI of a patient with SMA; Table S1: Correlation between body mass index, lipid metabolism and intramuscular fatty fraction in different muscle groups.

## Abbreviations

The following abbreviations are used in this manuscript:

CHOP-INTEND	Children’s Hospital of Philadelphia Infant Test of Neuromuscular Disorders
CSA	cross-sectional area
HINE	Hammersmith Infant Neurological Examination
HSFME	Hammersmith Functional Motor Score Expanded
IMFF	intramuscular fat fraction
MRI	magnetic resonance imaging
NPPV	non-invasive positive pressure ventilation
ROI	regions of interest
SMA	spinal muscular atrophy
