# Supplementary material for: Total Intramuscular Fat Fraction of Thigh Muscles as a Predictor of Nusinersen Efficacy in Pediatric SMA Type II and III
Source: Diagnostics (Basel). 2025 Mar 17;15(6):753. doi: 10.3390/diagnostics15060753 (PMC11941460; doi:10.3390/diagnostics15060753)
Supplement: Supplementary file 1 [file diagnostics-15-00753-s001.zip › diagnostics-3500217-supplementary.pptx]

## Slide 1
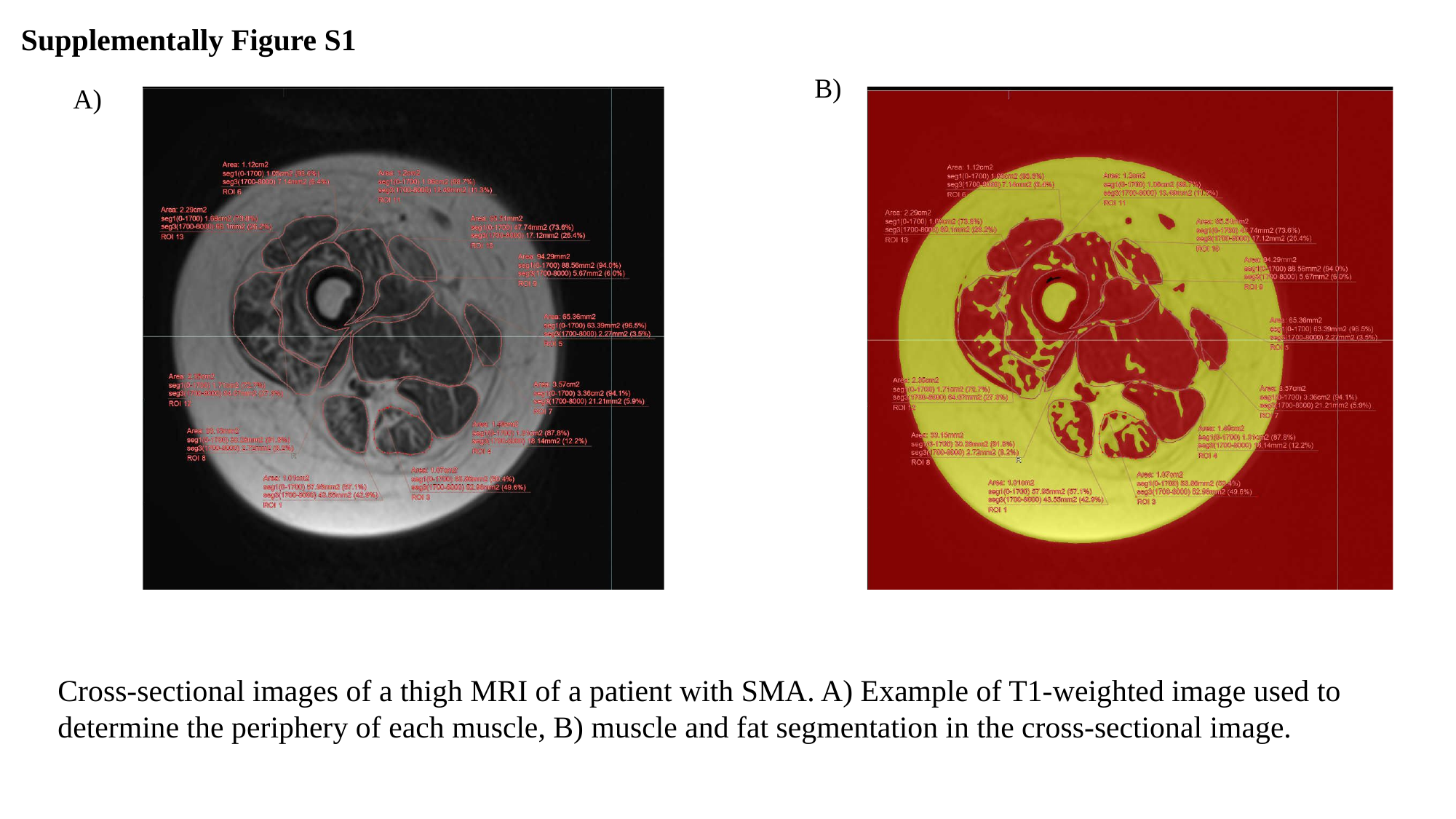

Supplementally Figure S1
B)
A)
Cross-sectional images of a thigh MRI of a patient with SMA. A) Example of T1-weighted image used to determine the periphery of each muscle, B) muscle and fat segmentation in the cross-sectional image.

## Slide 2
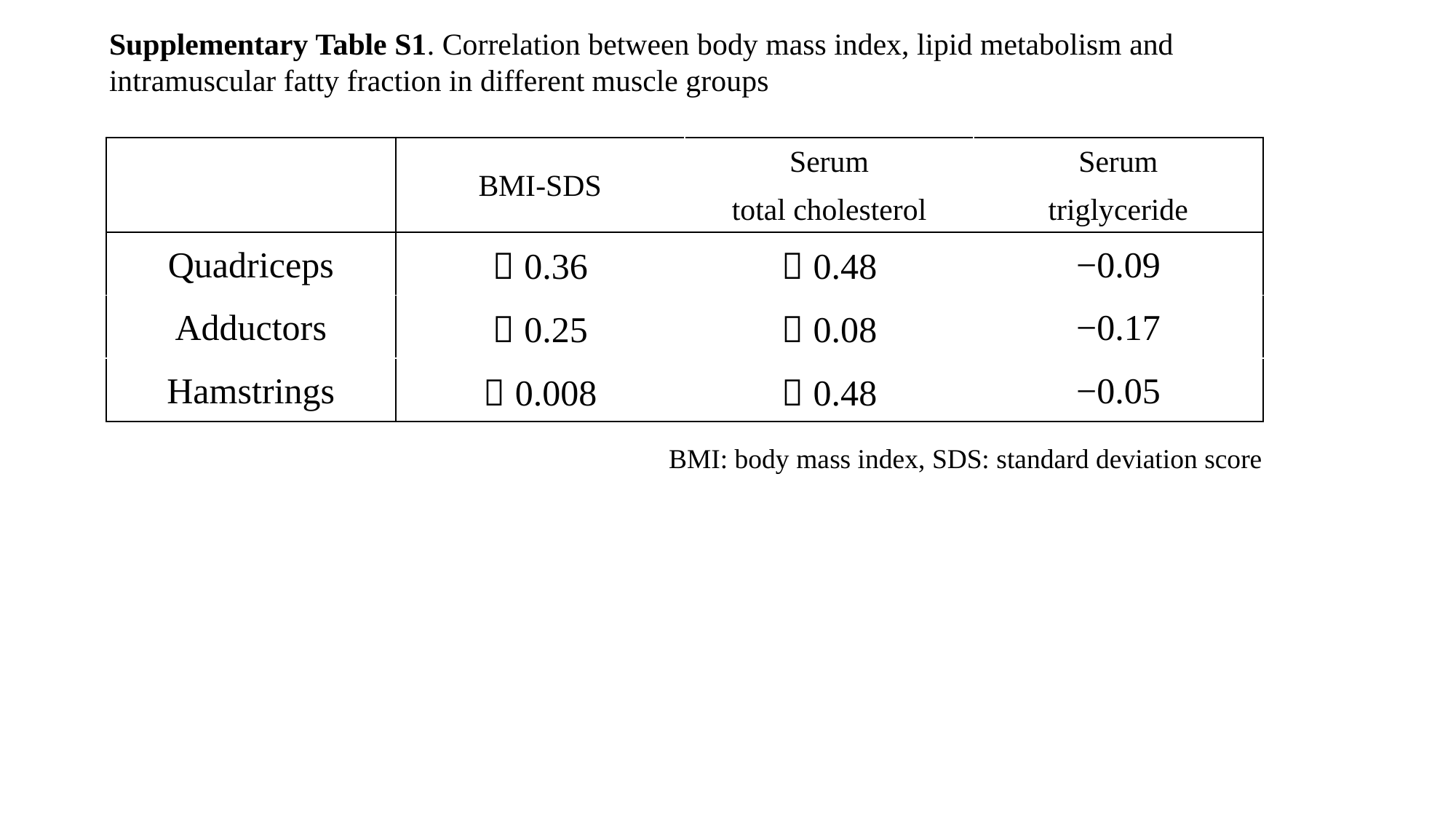

Supplementary Table S1. Correlation between body mass index, lipid metabolism and intramuscular fatty fraction in different muscle groups
| | BMI-SDS | Serum total cholesterol | Serum triglyceride |
| --- | --- | --- | --- |
| Quadriceps | －0.36 | －0.48 | −0.09 |
| Adductors | －0.25 | －0.08 | −0.17 |
| Hamstrings | －0.008 | －0.48 | −0.05 |
BMI: body mass index, SDS: standard deviation score
